# Barriers and facilitators to uptake of cervical cancer screening among women in Uganda: a systematic review

**DOI:** 10.1186/s12905-019-0809-z

**Published:** 2019-08-09

**Authors:** Eleanor Black, Fran Hyslop, Robyn Richmond

**Affiliations:** School of Public Health and Community Medicine, University of New South Wales, UNSW, Sydney, NSW 2052 Australia

**Keywords:** Cervical cancer, Cervical cancer screening, Barriers, Facilitators, Uganda

## Abstract

**Background:**

Uganda has one of the highest age-standardized incidence rates of cervical cancer in the world. The proportion of Ugandan women screened for cervical cancer is low. To evaluate barriers and facilitators to accessing cervical cancer screening, we performed a systematic review of reported views of Ugandan women and healthcare workers. The aim of this review is to inform development of cervical cancer screening promotional and educational programs to increase screening uptake and improve timely diagnosis for women with symptoms of cervical cancer.

**Methods:**

Fourteen studies that included the views of 4386 women and 350 healthcare workers published between 2006 and 2019 were included. Data were abstracted by two reviewers and findings collated by study characteristics, study quality, and barriers and facilitators.

**Results:**

Nineteen barriers and twenty-one facilitators were identified. Study settings included all districts of Uganda, and the quality of included studies was variable. The most frequently reported barriers were embarrassment, fear of the screening procedure or outcome, residing in a remote or rural area, and limited resources / health infrastructure. The most frequent facilitator was having a recommendation to attend screening.

**Conclusion:**

Understanding the barriers and facilitators to cervical cancer screening encountered by Ugandan women can guide efforts to increase screening rates in this population. Additional studies with improved validity and reliability are needed to produce reliable data so that efforts to remove barriers and enhance facilitators are well informed.

## Background

Cervical cancer (CC) is the most frequent cancer and the leading cause of cancer-related deaths among women in Uganda [1, 2]. Current estimates indicate that 6413 Ugandan women are diagnosed with CC annually, with 4301 deaths annually attributed to this disease [3]. Uganda has one of the highest incidence rates for CC in the world with an age-standardized rate of 54.8 per 100,000 women, compared with 6.6 in North America and 5.5 in Australia/New Zealand [3]. The age-standardized mortality rate in Uganda is 40.5 per 100,000 women, compared with an age-standardized mortality rate of 6.8 globally [3].

The most oncogenic types of Human Papillomavirus (types 16 and 18) are responsible for nearly all cases of CC. Human Papillomavirus (HPV) 16/18 prevalence among Ugandan women has been estimated at 33.6% [2], highlighting the importance of secondary prevention in this population. CC has a long pre-invasive phase, enabling detection of precancerous changes by screening before progression to invasive disease. While screening by cytology (‘Pap smears’) has prevented up to 80% of cervical cancers in high-resource settings [4], this approach is not currently feasible in Uganda due to inadequate infrastructure and lack of trained personnel [2]. Furthermore, the low sensitivity of cytology necessitates regular (2–3 yearly) screening intervals, which is problematic in Uganda because of poor follow-up and limited recall systems [2, 3, 5].

‘Screen-and-treat’ approaches using either HPV testing or visual inspection with acetic acid (VIA) followed by cryotherapy for precancerous lesions are a cost-effective prevention strategy in low-resource settings [6]. Guidelines for cervical cancer screening (CCS) in Uganda advocate a ‘see-and-treat’ approach where women aged 25 to 49 years are screened using VIA and treated with cryotherapy [7]. The guidelines recommend annual screening for HIV-positive women, and 3-yearly for all others, but in actuality screening is erratic and frequently determined by availability of resources. HPV testing has been shown in numerous studies to be extremely sensitive, and in research settings has been shown to be acceptable among Ugandan women [2]. However, it is currently limited to research settings and not yet widely available in Uganda [7].

While Uganda does not have a national CCS program, a key goal of Uganda’s national strategy for CC prevention and control is to have 80% of eligible women aged 25–49 years screened and treated for cervical precancerous lesions [7]. Baseline lifetime screening rate estimations are currently well below this target at between 4.8 to 30% [2, 8], and most women are diagnosed with advanced disease [2]. The combination of high HPV prevalence, low rates of CCS, and a paucity of cancer care facilities and specialists contributes to Uganda’s high mortality rate from CC [9]. The national CC prevention and control program has a focus on strengthening existing health systems to improve the accessibility of secondary prevention services [7]. Effective secondary prevention not only requires adequate infrastructure, but also acceptance and demand for screening by women and their communities [10]. Understanding factors that either encourage or inhibit women from engaging in CCS is critical to improving preventive strategies so as to reduce the incidence of invasive CC and its associated mortality. A small number of systematic reviews address barriers and facilitators to CCS uptake in Sub-Saharan Africa (SSA), but to the best of our knowledge this is the first systematic review focusing on this issue in Uganda.

This is a pressing public health issue, and has been identified as such by a number of recent articles calling for further research in this area [2, 11, 12]. CC affects women at the prime of their lives, with important social and economic consequences for their families and communities. Given that this is a largely preventable disease, the high incidence and mortality rates in Uganda are unacceptable. The purpose of this study is to [1] systematically review the current research on factors that may affect uptake of CCS among Ugandan women; and [2] draw well-informed conclusions that may be of use in shaping future public health efforts. Our results may inform the development of CCS promotional and educational programs to increase screening uptake among asymptomatic women and improve timely diagnosis for women with symptoms of cervical cancer.

## Methods

### Information sources and search

This systematic review was modelled on the PRISMA guidelines [13]. A systematic literature search was performed using Ovid MEDLINE, EMBASE, PsycINFO and SCOPUS in October 2017. The subject search and text word search were performed separately in all databases and then combined with ‘OR’ and ‘AND’ operators. The MeSH (Medical Subject Headings) terms included ‘cervical cancer’, ‘cervical neoplasms’, ‘cervical cancer screening’, ‘HPV testing’, ‘pap smear’, ‘visual inspection with acetic acid’, ‘barriers’, ‘facilitators’, ‘utilisation’, ‘Uganda’, ‘East Africa’, ‘Sub-Saharan Africa’. The search was limited to the year 1990 onwards and to English language, full-text articles. The database search was supplemented with searches on Google scholar, Proquest Theses and Dissertations database, manually examining reference lists of included articles and querying content experts. The last search was completed on 30th May 2019. The search outputs were saved where possible on databases and the authors received notification of any new searches meeting the search criteria.

### Data selection and synthesis

**Fig. 1:**
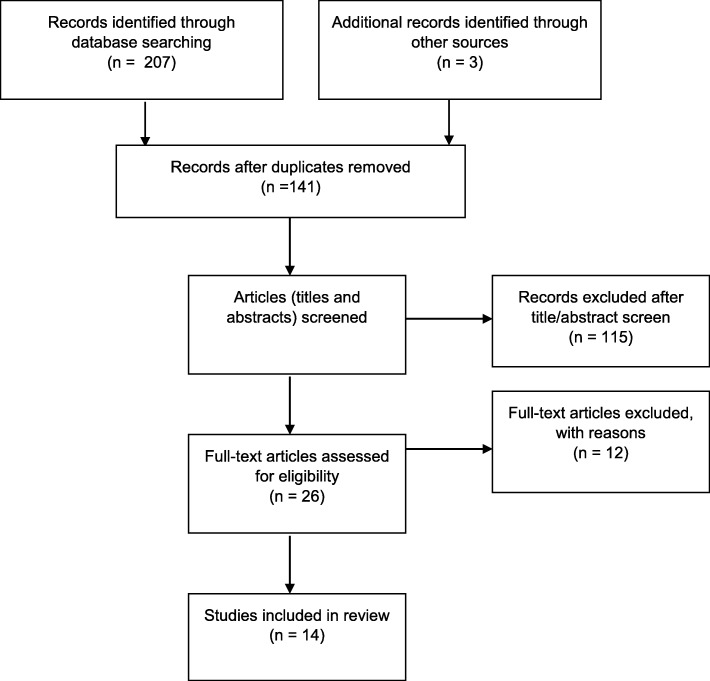
Diagram of Selection Process adapted from PRISMA Guidelines [13]

The initial database search returned 207 articles after removing unrelated titles, and 3 additional articles were identified through Google Scholar and from reference lists (see Fig. 1). The 69 duplicates were removed. The abstracts of the 141 articles were read and 115 studies excluded for not meeting the inclusion criteria. Following full-text review, 12 additional articles were excluded, as they did not specifically address barriers or facilitators to cervical cancer screening in Uganda. For example, some studies included data collected in Uganda as part of a larger study of African countries, but did not specify which findings were from Uganda.

Data were extracted from the remaining 14 papers independently by two of the authors (EB and FH). Discrepancies were resolved by discussion and consensus. For quantitative studies, data extracted included barriers and facilitators that were significantly associated with CCS intention or uptake, as well as proportions of participants reporting a barrier or facilitator. For qualitative studies, data extracted included all reported barriers/facilitators. Due to the heterogeneity in study designs, participants, and outcomes, a meta-analysis was not feasible. Instead, data from the studies was used to form a narrative analysis of barriers and facilitators to cervical cancer screening based on emergent themes.

### Eligibility criteria

Quantitative and qualitative studies examining barriers and/or facilitators to uptake of CCS among women in Uganda (any age) were included. Quantitative studies were included to identify associations between various factors and screening uptake, while qualitative studies were included to explore barriers and facilitators to screening that were reported by women or health care workers (HCWs). Studies that described the views of, or measured data from HCWs were included as it was anticipated they would have relevant insights into factors related to health systems and resources. Exclusion criteria were as follows: studies published prior to 1990, not in the English language or not available in full text, and those that did not specifically address barriers or facilitators to uptake of cervical cancer screening among Ugandan women. Studies that focused on barriers faced by women with HIV were not included given that this group of women face their own, unique challenges to accessing screening services [14].

### Quality assessment and analysis

Included studies were subjected to a quality assessment using an appraisal method designed and evaluated by Sirriyeh and colleagues for use in studies with diverse designs [15]. The tool uses a 16-item scale with a 4-point scoring system and allows for an assessment of the overall quality of mixed qualitative and quantitative data. Given the small number of included studies, no studies were excluded based on their quality score.

## Results

### Study characteristics

**Fig. 2:**
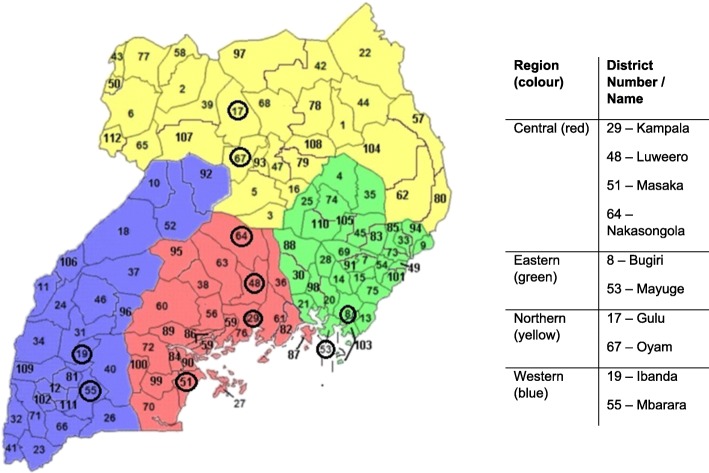
Districts of Uganda represented by included studies Source: adapted from Districts of Uganda, Wikipedia

Overall, 14 studies were included in the final analysis. Eight were cross sectional, five were qualitative studies using focus group discussions (FGDs) and key informant interviews (KIIs), and one was a mixed methods study. Table 1 provides information on the author, publication year, region/study site, sample size, research methods, and the type of statistical analysis used for quantitative studies. It also specifies which type of CCS (if relevant) was addressed in the study, and what proportion of women in the study had been screened (if measured). The 14 studies were published between 2006 and 2017 and comprise of a mix of urban and rural study populations, with at least two districts per region represented (see Fig. 2 below). Five studies focused on visual inspection methods (VIA/VILI), two on HPV self-collection, one on cytology, and six looked generally at screening without specifying a particular screening method. The studies covered a total of 4386 women and 350 HCWs. The proportion of women ever screened was measured in six studies and ranged from 4.8 to 35.1%. The highest screening rates were found among studies recruiting from women already attending health clinics [16, 17], and consequently these findings are not representative of the Ugandan population. These higher rates possibly reflect the recruitment and sampling design of these studies, whereby participants may have been encouraged and/or referred by HCWs to attend the health clinics. In studies conducted at the household level [8, 18, 19] and where multi-stage sampling was used [8, 18], the proportion of women screened was lower.

**Table 1:** Characteristics of Included Studies

Authors/ year	Region/ study site	Sample size	Study design/ instrument	Screening type	Proportion of women ever screened	Statistical Analysis
Busingye 2012	Mulago Hospital, Kampala	384 women, age not reported	Mixed methods / interviewer-administered questionnaires, FGDs	VIA/ VILI	Not measured	Descriptive and bivariate
Hasahya 2016	Nakasongola & Ibanda districts	36 women aged 25–49	Qualitative / FGDs	N/A	Not measured	N/A
Li 2017	Luweero district	625 women, age not reported	Cross-sectional / interviewer-administered questionnaires	VIA	Not measured	Descriptive and multivariate
Mitchell 2011	Kisenyi district, Kampala	300 women aged 30–65	Cross-sectional / interviewer-administered questionnaires	HPV self-collection	8%	Descriptive and multivariate
Mutyaba 2006	Mulago Hospital, Kampala	285 HCWs	Cross-sectional / self-administered questionnaires	Papsmear	N/A	Descriptive
Mwaka 2013	Gulu district	15 HCWs	Qualitative / key informant interviews (KII)	VIA/ VILI	Not measured	N/A
Ndejjo 2017a	Bugiri & Mayuge districts	900 women aged 25–49	Cross-sectional / interviewer-administered questionnaires	N/A	4.8%	Descriptive and multivariate
Ndejjo 2017b	Bugiri & Mayuge districts	119 (107 women, 12 HCWs)	Qualitative / FGDs and KIIs	N/A	Not measured	N/A
Ndejjo 2016	Bugiri & Mayuge districts	900 women aged 25–49	Cross-sectional / interviewer-administered questionnaires	N/A	4.8%	Descriptive and multivariate
Osingada 2015	No-cost reproductive clinic (location not disclosed)	236 women aged 18 and over	Cross-sectional / interviewer-administered questionnaires	VIA/ VILI	28.8%	Descriptive and multivariate
Paul 2013	Nakasongola, Mbarara,Ibanda districts	53 (21 women, 32 HCWs)	Qualitative / KIIs and FGDs	VIA	Not measured	N/A
Teng, 2014	Primary & tertiary setting, Kampala	22 (6 HCWs, 16 women aged 30–69)	Qualitative / KIIs and FGDs	HPV self-collection	Not measured	N/A
Twinomujuni 2015	Masaka district	416 women aged 25–49	Cross-sectional / interviewer-administered questionnaires	N/A	7%	Descriptive and multivariate
Waiswa 2017	Oyam district	445 women aged 15–49	Cross sectional / interviewer-administered questionnaires	N/A	35.1%	Descriptive and bivariate

### Study quality assessment

Table 2 summarizes the quality assessment findings. Scores for quantitative studies ranged from 11 to 40, and qualitative scores ranged from 12 to 39. Four studies based their investigation on an applied theoretical framework. All studies gave a clear description of the research setting and 12 of the studies completely identified their objectives. The sample was broadly representative of the target population in seven studies. Data collection procedures were described well by six studies although among the quantitative studies only a few reported assessment of reliability and validity of the survey tool. Most studies provided a fair explanation of their choice of analysis method. A few of the qualitative studies used a range of methods to assess reliability, but two studies did not report on this item.

**Table 2:** Quality assessment using the tool developed by Sirriyeh et al. for diverse study designs

Author, Year	1	2	3	4	5	6	7	8	9	10	11	12	13	14	15	16	Total Score
Busingye, 2012	2	3	3	0	2	2	3	3	0	2	3	2	0	0	0	2	27
Hasahya, 2016	2	3	3	3	2	3	3	3	n/a	n/a	3	3	3	3	3	2	39
Li, 2017	0	3	3	0	2	2	0	3	0	2	n/a	3	1	n/a	0	1	20
Mitchell, 2011	3	2	3	0	2	2	3	2	2	2	n/a	3	2	n/a	2	2	30
Mutyaba, 2006	0	1	3	0	2	1	1	0	0	2	n/a	1	0	n/a	0	0	11
Mwaka, 2013	0	3	3	3	1	3	2	3	1	1	n/a	3	2	n/a	2	2	29
Ndejjo 2017a	0	3	3	3	3	1	1	3	1	2	n/a	3	2	n/a	1	2	28
Ndejjo 2017b	0	3	3	2	1	3	2	3	n/a	n/a	2	2	1	3	1	3	29
Ndejjo 2016	0	3	3	3	3	1	1	3	1	2	n/a	3	2	n/a	1	3	29
Osingada 2015	3	3	3	3	3	3'	3	3	1	2	n/a	3	1	n/a	3	3	37
Paul 2013	0	3	3	0	2	1	0	0	n/a	n/a	2	1	0	0	0	0	12
Teng 2014	3	3	3	0	2	3	3	3	n/a	n/a	3	3	2	3	2	3	36
Twinomujuni 2015	3	3	3	3	2	3	3	3	3	2	n/a	3	3	n/a	3	3	40
Waiswa 2017	0	3	3	3	2	1	1	2	0	2	n/a	2	1	n/a	2	3	25

Criteria (scoring items as 0 = not at all, 1 = very slightly, 2 = moderately, 3 = complete)

1 = explicit theoretical framework; 2 = statement of aims/objectives in main body of report; 3 = clear description of research setting; 4 = evidence of sample size considered in terms of analysis; 5 = representative sample of target group of a reasonable size; 6 = description of procedure for data collection; 7 = rationale for choice of data collection tool(s); 8 = detailed recruitment data; 9 = statistical assessment of reliability and validity of measurement tool(s) (quantitative only); 10 = fit between stated research question and method of data collection (quantitative); 11 = fit between stated research question and format and content of data collection tool (e.g., interview schedule) (qualitative); 12 = fit between research question and method of analysis; 13 = good justification for analytical method selected; 14 = assessment of reliability of analytical process (qualitative only); 15 = evidence of user involvement in design; 16 = strengths and limitations critically discussed

### Analysis of included studies

19 barriers and 21 facilitators emerged from thematic analysis. The number and type of studies in which these were reported is summarized in Table 3. For quantitative studies, a distinction is made between those studies that reported proportions/other results, and those that identified statistically significant associations. Barriers reported by the greatest number of studies were embarrassment, fear of the screening procedure, fear of outcome, residing in a remote or rural area, limited resources/health infrastructure, and limited access to screening care. Being recommended to attend screening was the facilitating factor most consistently reported across studies. One barrier was statistically significant and this was having limited access to CCS facilities. Knowledge of CCS, perceiving oneself as at risk of CC, and being recommended to attend screening were statistically significant facilitators in two studies each. Because of the wide range in methodologies, sample sizes and study scopes of the included studies, it is not possible to draw conclusions about the most significant barriers or facilitators. Nevertheless, these results illustrate that certain factors have been identified as important barriers or facilitators by numerous studies. These factors merit further evaluation by future studies.

**Table 3:** Barriers and Facilitators to uptake of CCS by study design

Barriers	Identified as statistically significant in QN study (# studies)	Identified as proportion or other result in QN study (# studies)	Identified in a QL study (# studies)	Total # studies in which identified
Poor knowledge of CC	_	_	1. Hasahya 2016 2. Mwaka 2013 3. Ndejjo 2017b 4. Teng 2014	4
Poor knowledge of CCS	_	_	1. Hasahya 2016 2. Mwaka 2013 3. Ndejjo 2017b	3
Low perceived risk of CC	_	1. Mutyaba 2006 2. Ndejjo 2017a 3. Twinomujuni 2015	_	3
CC not considered significant / CCS not considered important	_	1. Twinomujuni 2015 2. Waiswa 2017	1. Teng 2014	3
Embarrassment	_	_	1. Hasahya 2016 2. Mwaka 2013 3. Ndejjo 2017b 4. Paul 2013 5. Teng 2014	5
Lack of privacy	_	1. Busingye 2012 2. Teng 2014	1. Mitchell 2011 2. Twinomujuni 2015	4
Fear of screening	_	1. Li 2017 2. Twinomujuni 2015	1. Busingye 2012 2. Hasahya 2016 3. Mwaka 2013 4. Paul 2013 5. Teng 2014	7
Fear of outcome	_	_	1. Busingye 2012 2. Hasahya 2016 3. Ndejjo 2017b 4. Paul 2013 5. Teng 2014	5
Lack of financial / emotional support from spouse	_	_	1. Mwaka 2013	1
Stigma	_	_	1. Busingye 2012 2. Hasahya 2016 3. Ndejjo 2017b 4. Teng 2014	4
Traditional healers accessed over HCWs	_	_	1. Ndejjo 2017b	1
Older age	_	1. Mitchell 2011	_	1
Residing in a remote or rural area	_	1. Waiswa 2017	1. Hasahya 2016 2. Mwaka 2013 3. Ndejjo 2017b 4. Paul 2013	5
Limited access to CCS facility	1. Ndejjo 2016	1. Osingada 2015 2. Waiswa 2017	1. Mwaka 2013 2. Ndejjo 2017b	5
Limited resources and health infrastructure	_	1. Mutyaba 2006	1. Hasahya 2016 2. Mwaka 2013 3. Ndejjo 2017b 4. Paul 2013	5
No time / long wait times	_	1. Li 2017	1. Busingye 2012 2. Paul 2013	3
Perceiving HCWs as rude	_	_	1. Ndejjo 2017b	1
Lack of trained HCWs	_	_	1. Mwaka 2013	1
Financial costs associated with CCS	_	1. Twinomujuni 2015	1. Mwaka 2013 2. Ndejjo 2017b 3. Paul 2013	4
Facilitators	Identified as statistically significant in QN study (# studies)	Identified as proportion or other result in QN study (# studies)	Identified in a QL study (# studies	Total # studies in which identified
Knowledge of CC	_	_	1. Ndejjo 2017b 2. Teng 2014	2
Knowledge of CCS	1. Ndejjo 2016 2. Ndejjo 2017a	_	_	2
Perceived risk of CC	1. Mitchell 2011 2.Twinomujuni 2015	1. Ndejjo 2017a	_	3
CC considered significant disease / CCS considered important	_	1. Ndejjo 2017a	_	1
Experiencing signs / symptoms of CC	–	1. Ndejjo 2016	1. Ndejjo 2017b 2. Paul 2013	3
Fear of outcome	_	_	1. Paul 2013	1
Not afraid of outcome	1. Twinomujuni 2015	_	_	1
Wanted to know health status	_	1. Ndejjo 2016 2. Ndejjo 2017a	1. Ndejjo 2017b	3
Family or spousal support	_	1. Twinomujuni 2015	1. Paul 2013	2
Personal / family experiences with CC or CCS	1. Ndejjo 2016	_	1. Hasahya 2016 2. Ndejjo 2017b	3
Recommended to attend screening	1, Ndejjo 2016 2. Osingada 201	1. Twinomujuni 2015	1. Mwaka 2013 2. Paul 2013	5
Age > 25 years	1. Osingada 2015	_	_	1
Postsecondary or greater education	1. Busingye 2012	_	_	1
Higher income	1. Ndejjo 2017a	_	_	1
Formal employment	1. Twinomujuni 2015	_	_	1
Living with spouse	1. Twinomujuni 2015	_	_	1
Smaller household size	1. Ndejjo 2016	_	_	1
Residing in urban or semi urban areas	1. Ndejjo 2016	_	_	1
Access to health facility where CCS offered	_	1. Ndejjo 2017a	_	1
Not being concerned about gender of HCW	1. Osingada 2015	_	_	1
Community Outreach	1. Osingada 2015	_	_	1

*QN = quantitative study QL = qualitative study*

### Barriers & Facilitators: individual, social and structural factors

Barriers and facilitators were categorized into three main categories for the purpose of this review: individual, sociocultural, and structural factors.

- Individual Factors

### Knowledge of CC/CCS

Poor knowledge of CC was a barrier in four qualitative studies, and poor awareness of CCS was a barrier in three qualitative studies. Some women did not know about the cause of CC and many women did not know of any screening method. HCWs felt that low screening uptake could be attributed to poor knowledge of CC. Conversely, adequate knowledge of at least one screening method was significantly associated with having been screened [8] or having intention to screen [18] in two quantitative studies. However, it is not possible to ascertain the direction of causality and it is possible that women are knowledgeable as a result of having been screened rather than it being the reason for screening.

### Perceived risk and importance of CC/CCS

In three quantitative studies women with low risk perception were less likely to report intention to screen [11, 18, 19]. Some women had not been screened because they believed it was unnecessary in the absence of symptoms. Conversely, women who felt at risk were twice as likely to report intention to screen [19], and feeling at risk was significantly associated with willingness to collect an HPV sample [20].

### Experiencing CC signs/symptoms

Experiencing signs and symptoms of CC was a trigger to seeking CCS amongst women in two qualitative studies [12, 21] and one quantitative study [8]. Ndejjo and colleagues reported that signs and symptoms were the strongest trigger to accessing CCS among the women in their study [12].

### Embarrassment

Five qualitative studies reported that embarrassment related to the intimate nature of VIA/pap smears was a deterrent to screening. Self-collected HPV testing was regarded as embarrassing by women in one qualitative study [22]. Location of screening and whether privacy was afforded also affected willingness to screen in two quantitative and two qualitative studies. Importantly, Teng and colleagues found that women universally agreed that embarrassment would not be a major deterrent to screening if they were well informed about the need to screen, and if a private place for self-collection of HPV swabs was available [22].

### Fear of screening procedure

Five qualitative and two quantitative studies reported on fear related to the screening procedure. In many cases this related to fear of pain. Fear of becoming infected through non-disposable speculums or poor sanitary practices was reported in three qualitative studies [14, 21, 23]. Fear that the procedure might cause cancer [22], lead to ‘enlargement of the sexual parts’, [23] or ‘pull out the uterus’ [21] were also reported.

### Fear of results/fatalism

Fear of being diagnosed with CC, often coupled with a sense of fatalism regarding prognosis and implications, was a reported barrier in five qualitative studies. Notably, in Paul et al’s qualitative study, fear of receiving a CC diagnosis motivated some women to attend screening [21]. Women who reported being unafraid of receiving a diagnosis were significantly more likely to have intention to screen in one study [19].

b) Social and Cultural Factors

### Gender power relations

In one quantitative study, HCWs reported that lack of spousal emotional and financial support was a barrier to CCS [24]. Conversely, women in Teng et al’s qualitative study universally stated that they would attend CCS regardless of whether or not their spouse approves [22], and spousal approval did not influence women’s willingness to self-collect HPV samples in Mitchell et al’s cross sectional study [20].

### Family / spousal support

Encouragement from family members to attend screening, particularly spousal encouragement, was an important motivator for women in Paul et al’s qualitative study [21]. Women who reported discussions with their husbands about screening were more likely to report intention to screen in one quantitative study [19].

### Stigma

Concern about how screening was perceived by community members and family was a barrier reported by four qualitative studies. A common preoccupation was that CCS might also reveal one’s HIV status, leading to societal rejection. In one qualitative study women were concerned their spouse might leave them if they were found to have CC due to resultant treatment expenses [12].

### Personal or family experiences with CC / CCS

Having known somebody with CC, or somebody who had undergone CCS, was a motivating factor for women to access screening in one quantitative and two qualitative studies. Some women related that loss of a family member to CC had motivated them to be screened [12, 14]. In one cross sectional study, women who knew someone who had ever been screened or diagnosed were significantly more likely to have been screened [8].

### Recommended for CCS

Being recommended to attend screening by HCWs was a significant facilitator in Ndejjo et al’s study, where women who had been recommended by a HCW were 87 times more likely to have been screened for CC compared with their counterparts [8]. Osingada et al. found that women who had never received encouragement to screen from HCWs were 84% less likely to have been screened [16].

### Traditional healers

In one study, several HCWs reported that many women first seek healthcare from traditional practitioners because of the perception that CC is caused by witchcraft. This was described as being a barrier to CCS in that it delays screening among women who first look for traditional cures [12].

c) Structural Factors

### Socioeconomic and demographic conditions

In one study, women with postsecondary education were significantly more likely to have been screened than their less educated counterparts [23]. Formal employment was seen to significantly facilitate screening [19], and women whose households earned more than 40 US dollars per month had a significantly higher level of intention to screen [18]. In one cross sectional study, respondents who lived in households with five or less members were twice as likely to have undergone CCS compared with their counterparts [8]. Living in a remote or rural area was a barrier to screening in four qualitative and one quantitative study.

### Access to CCS

Women found it difficult to present for screening when health facilities were not nearby, as reported in three quantitative and two qualitative studies. Waiswa and colleagues found that 32.9% of the women who had never been screened attributed this to not having a nearby facility [17]. Ndejjo et al. found that women who lived within a 5 km radius of a health facility where CCS was offered had a higher intention to screen [18].

### Limited resources / infrastructure

Four qualitative and one quantitative study reported staffing shortages, lack of pathology services and limited health infrastructure as barriers to provision of CCS [11, 12, 14, 21, 24]. Lack of speculum equipment in some cases meant that women who presented for screening had to be turned away [21].

### Time constraints

Time constraints and prohibitively long waits at health facilities were barriers in one quantitative and two qualitative studies. In Li et al’s cross sectional study, 27.8% of the women who refused screening did so because of time constraints [25].

### HCW qualities

Women in Ndejjo et al’s qualitative study reported that rude or insensitive HCWs were a disincentive to attend screening [12]. In one quantitative study, women who were not concerned about the gender of the HCWs performing the screening were 5 times more likely to have been screened compared with those who were [16]. HCWs reported that lack of training and skills for CCS among some of the clinical staff was a barrier to provision of CCS [24].

### Costs related to CCS

Financial costs associated with screening were a barrier for women in four included studies, and related either to the cost of the service or to associated transport/food costs [12, 19, 21, 24]. Twinomujuni et al. found that total costs for services were reported as prohibitive by 89.7% of the women in their survey [19].

### Community outreach services for CCS

In one quantitative study, women who had attended community outreach services for CCS were significantly more likely to have engaged with screening services [16]. There was no reference to outreach services in any of the other included studies.

## Discussion

Women and HCWs in Uganda identified a number of barriers and facilitators to uptake of CCS. These act at multiple levels (individual, sociocultural, and structural) and were similar across districts.

The most commonly reported barrier was fear of the screening procedure. This was often related to perceived pain, but also to misconceptions including that infected equipment might be used or vital organs removed. Fear of being diagnosed with CC, coupled with a sense of fatalism, was another reported barrier. While this is somewhat understandable given the high mortality rate from CC in Uganda, women were generally uninformed about the role of screening in identifying and controlling early disease, and many believed screening was unnecessary in the absence of signs or symptoms. Hence poor knowledge of CCS, which was another commonly reported barrier, likely exacerbates these misconceptions and fears.

Women in the surveys explicitly stated that improved knowledge of CC would help them to understand the benefits of screening, and some reported that messages about CCS on the radio or at health facilities had motivated them to be screened [12]. Communication about the need for screening is a key area of need identified by this review. However, improved knowledge alone is unlikely to be sufficient; one of the studies demonstrated that uptake of CCS among medical workers was low, signaling that even among those who are presumably well informed about the benefits of screening, additional barriers to care exist.

Embarrassment related to the screening procedure was another commonly reported barrier. Given the nature of the screening procedure this is a difficult barrier to remove, however it can be ameliorated by ensuring privacy and having female HCWs available at facilities. HPV self-collection is a promising means of overcoming embarrassment and obviates the need for HCWs to be female. Although an included study reported that women found self-collection for HPV embarrassing, this is in discordance with previous reviews that have reported high acceptance of this screening method among Ugandan women and women in low-resource settings [2, 26, 27]. Encouragingly, this review also found that embarrassment about the procedure is not static and can be reduced through improved knowledge of the need for screening. Thus, efforts to improve knowledge about CC would likely help women to overcome the embarrassment barrier.

Generally, structural factors associated with screening uptake were not surprising. Lack of adequate health infrastructure and resources is a well-recognized barrier to screening in Uganda and was reported as such by most studies. Beyond being a barrier to screening, inadequate health infrastructure may negate the effect of increased uptake of CCS, as diagnostic and treatment capacity needs to be able to meet any increased demand created as a result of screening. The impact of health system factors in reducing the CC burden in Uganda was beyond the scope of this review, but is an important topic that deserves further research.

This review found that lower levels of income and education along with lack of formal employment and larger household sizes were barriers to screening. Socioeconomic and demographic inequalities have profound influences on health-seeking behaviours, and relate significantly to high CC incidence and mortality rates [28]. Many studies in this review reported that accessing screening was more difficult for women living in rural/remote regions. Special efforts must be made to facilitate these women, for example via mobile health units with availability of screen-and-treat facilities.

In contrast to other studies in SSA, women in these studies indicated that lack of spousal support was not a barrier to accessing screening. However, a number of women were concerned their spouse might leave them or refuse to pay for care if they received a diagnosis of either CC or HIV, indicating that gender power relations were influential at some level. Previous studies have reported that gender power relations in Uganda are patriarchal, with men traditionally controlling family finances and access to health services [24, 29]. Interestingly, this was only reflected in one of the included studies [24]. Although the data from this review was inadequate to draw strong conclusions on the role of men in influencing uptake of CCS, involving men in the screening process may be beneficial both in facilitating women to attend (through emotional and financial support), and in ensuring follow up. An RCT from Uganda demonstrated that among women referred for colposcopy following a positive screening test, those whose spouses were involved were more likely to return for colposcopy [29].

Importantly, women and HCWs in the included studies identified a number of facilitators to CCS. For many women, encouragement to attend screening, by HCWs or other women, was a key facilitator. This was statistically significant in two studies, and infers that health promotion by trusted community members enabled women to overcome other barriers. Sadly, despite CC being the number one cause of cancer incidence and mortality among Ugandan women, a large number of women in the studies considered that CC was not an important issue. This may reflect ineffective health promotion messages and/or a perceived unimportance of the issue relative to other commitments and responsibilities. HCWs should be encouraged to ask and make recommendations about screening opportunistically, at every health meeting. Attendance at a community outreach service for CCS was a motivator for women to attend CCS in one of the studies [16], and may be another useful strategy for informing and engaging women.

In two of the studies that offered VIA/VILI to recruited women, acceptance rates were high (> 90%) [23, 25]. This may reflect that the act of being invited to partake in screening was in itself a facilitator and that, similar to encouragement, may be a strategy that HCWs could employ. Another possible reason for the high acceptance rate in these studies was that women were already in a healthcare setting (immunization clinic or outpatient department), so the costs involved in reaching a healthcare setting had already been overcome. Time constraints and financial barriers were reported by women in a number of included studies. Integration of CCS with reproductive and maternal health services, such as postnatal or HIV clinics, may help overcome these logistical barriers. Although attendance at postnatal and immunization clinics in Uganda is also low, integration of services would conceivably improve attendance by removing the need for multiple, costly trips and creating a ‘one-stop shop’.

### Strengths and limitations of this review

To our knowledge, this is the first systematic review to focus on barriers and facilitators to uptake of CCS among women in Uganda. Data on factors that enable women to access screening is required to provide information about how CCS uptake may be improved and is of particular importance given that CCS uptake in Uganda is low in the setting of high CC and HPV incidence. This review focuses on the views of women as well as HCWs and thus contributes valuable information regarding the perspective of the target group for screening, as well as insights from professionals who provide this care. The collective evidence may guide the development of health promotion programs that incorporate the views of the target group.

While this review found general agreement among the HCWs and women in the included studies, and between women living in different regions, the small number of included studies limited a deeper understanding of district-specific barriers or facilitators. For example, post-conflict Northern Uganda has a large proportion of internally displaced women who likely have different competing priorities and may face different barriers to women in other districts. The small number of studies included in the review also meant that some barriers/facilitators were not identified. Furthermore, questionnaire types were often pre-established questions determined by the investigators, and some studies did not provide details regarding questions asked or themes explored. Depending on the way that questions were structured, relevant barriers and facilitators may not have emerged.

The quality of included studies was highly variable. In some studies the investigation of barriers or facilitators was not the primary outcome of the study. More studies specifically designed to address barriers and facilitators among women in Uganda are needed. Statistical assessment of reliability and validity of measurement tools was not at all evident, or only slightly evident, in eight of the quantitative studies. This limits the quality of findings from this review and signals a need for more rigorous study design in future studies.

## Conclusion

This review has presented the perspectives of women and HCWs on the factors that enable or hinder women in Uganda from seeking and accessing CCS. We have found that important barriers include fear of the procedure and outcome, embarrassment, stigma, living in rural or remote regions with limited access to screening services, low levels of knowledge of CC, and low perceived risk. We found that encouragement to attend screening by other women or HCWs is an important facilitator to accessing screening, and perceived personal risk of CC was also influential in the decision to screen. The findings from this review illustrate the complex interplay of individual, sociocultural and structural barriers that prevent women from accessing CCS in Uganda, and also highlight key enabling factors. The sociocultural factors that influence CCS in Uganda appear to be influential and suggest that community input will be essential to implementing effective change. Local women community leaders and champions will likely be key to informing women on the need for screening and hence increasing the demand for services. Our results should be interpreted and applied judiciously, given the limitations identified above.

## Data Availability

The datasets used and/or analysed during the current study are available from the corresponding author on reasonable request.

## Abbreviations

CC: Cervical cancer
CCS: Cervical cancer screening
FGDs: Focus group discussions
HCW: Health care worker
HPV: Human Papillomavirus
KIIs: Key informant interviews
SSA: Sub-Saharan Africa
VIA: Visual inspection with acetic acid
VILI: Visual inspection using Lugol’s iodine
